# Relationship between body segment movements and center of pressure shifts during trunk lean movements while sitting in healthy adults

**DOI:** 10.3389/fresc.2026.1837819

**Published:** 2026-06-10

**Authors:** Boqun Liu, Shintaro Uehara, Akiko Yuasa, Hirofumi Ota, Kazuki Ushizawa, Yohei Otaka

**Affiliations:** 1 Department of Rehabilitation Medicine, Fujita Health University Graduate School of Medicine, Aichi, Japan; 2 Department of Rehabilitation Medicine, Shengjing Hospital of China Medical University, Liaoning, China; 3 Faculty of Rehabilitation, Fujita Health University School of Health Sciences, Aichi, Japan; 4 Department of Rehabilitation Medicine, Fujita Health University School of Medicine, Aichi, Japan; 5 Japan Society for the Promotion of Science, Tokyo, Japan

**Keywords:** center of pressure, motion capture, pelvis, postural balance, reliability, sitting position

## Abstract

**Introduction:**

Sitting activities involve trunk leaning, which requires precise coordination of the head, trunk, and pelvis to maintain the center of pressure (COP) within limits of stability. However, how the movement in each segment is associated with COP displacement remains unclear.

**Methods:**

Twenty-eight healthy volunteers performed seated trunk leaning twice in left, right, forward, and backward directions. COP displacements and angular changes were measured in head, trunk, and pelvic tilt in all directions, and additionally in the hip during forward-backward leaning. Within-session reliability of measured indices was assessed using intraclass correlation coefficients (ICCs) and Bland-Altman analyses, and relationships between segment angle changes and COP displacements were examined using mixed-effects models.

**Results:**

ICCs between two trials showed substantial to very high correlations (ICC = 0.84-0.95), and Bland-Altman analyses showed neither fixed nor proportional bias, suggesting sufficient reliability. Pelvic tilt angle changes were significantly associated with COP displacements during left-right and forward-backward leaning (*p* < 0.001). Head, trunk, and hip angle changes were also significantly associated with COP displacement during forward-backward leaning (all *p* < 0.01).

**Conclusions:**

Pelvic tilt was associated with COP displacement during leaning, regardless of direction, indicating the importance of pelvic shifts in strategies for maintaining sitting balance in healthy adults.

## 1 Introduction

Sitting balance ability is essential for daily activities, such as eating, dressing, grooming, and reaching objects, as it helps maintain posture and protects against falls (1). However, individuals with disabilities, such as those who experienced stroke and spinal cord injury, as well as older adults, may have problems with sitting balance because of motor and sensory impairments and musculoskeletal problems (2, 3). Reduced balance control leads to an abnormal strategy of body movements while performing activities in the sitting position compared to the strategy of healthy individuals (4–6). Moreover, postural control during sitting may partially share control mechanisms with standing (7). This broader relevance is clinically important because impaired standing balance has been associated with future fall risk (8) and all-cause mortality (9). Therefore, clarifying postural control during sitting may also provide a basis for understanding motor control mechanisms of postural balance.

The center of pressure (COP), which is the center of the distributed force applied to the supporting surface (10), is a verified, valid, and reliable indicator for objectively assessing sitting balance ability (5, 11, 12). In seated activities of daily living, such as putting on shoes, washing the feet, and reaching for objects, individuals often need to lean the upper body and maintain dynamic control of COP within the limits of stability. Thus, seated trunk leaning can be regarded as a functional, multi-segment movement mainly involving the head, trunk, and pelvis that reflects dynamic aspects of sitting balance control. During such movements, the maximal distance an individual can voluntarily move their COP without losing balance is one parameter that specifies sitting balance ability, based on the notion that this measure accounts for dynamic balance while standing (13, 14). Previous studies have suggested that the coordinated movements of individual body segments, including the head, trunk, and pelvis, contribute to the voluntary, dynamic control during seated leaning, especially reaching distance (4, 15). In addition, reaching distance has been reported to be associated with COP displacement (16). Thus, the coordinated segment movements may be related to how far the COP can be displaced while sitting. Nevertheless, the manner and extent to which changes in segmental movements are associated with COP displacements across different leaning directions in healthy adults remains unclear. This healthy normative baseline provides a quantitative reference for interpreting pathological compensatory strategies and supports the development of clinical protocols and the evaluation of interventions for older adults and neurological populations.

To address this question, the present study investigated the relationship between body segment movements (i.e., changes in body segment angles) and COP displacement during trunk leaning while sitting in healthy individuals.

## 2 Methods

### 2.1 Participants characteristics

We aimed to recruit 30 healthy adults for this study, referring to previous reliability studies and seated reaching/trunk-leaning COP studies that commonly included approximately 20–40 participants (11, 16–20). Participants were recruited between August 2023 and January 2024. The inclusion criteria were no current musculoskeletal pain, injury, or functional limitation of the trunk or lower limbs that could affect seated trunk leaning or sitting balance. The exclusion criterion was a history of neurological or neuromuscular disease. However, one participant was excluded from the analysis due to experimental error, and another withdrew due to back pain experienced during the task. Therefore, 28 participants (14 females; mean age: 22.6 years, range 21–31 years) were included in the final analysis (Table 1). All participants provided written informed consent. The study was performed in accordance with the 2013 revision of the 1964 Declaration of Helsinki. The Ethics Review Committee of Fujita Health University approved this study (approval no. HM23-233).

**Table 1:** Participant characteristics.

Characteristic	Mean (standard deviation)
Age, years	22.6 (2.4)
Sex, male/female, n	14/14
Height, cm	164.2 (8.6)
Body mass, kg	55.6 (7.6)
Body mass index, kg/m^2^	20.6 (1.8)
Thigh length^a^, cm	40.3 (2.3)

a The distance between the right greater trochanter and the right lateral epicondyle of the femur.

### 2.2 Experimental procedure

**Figure 1:**
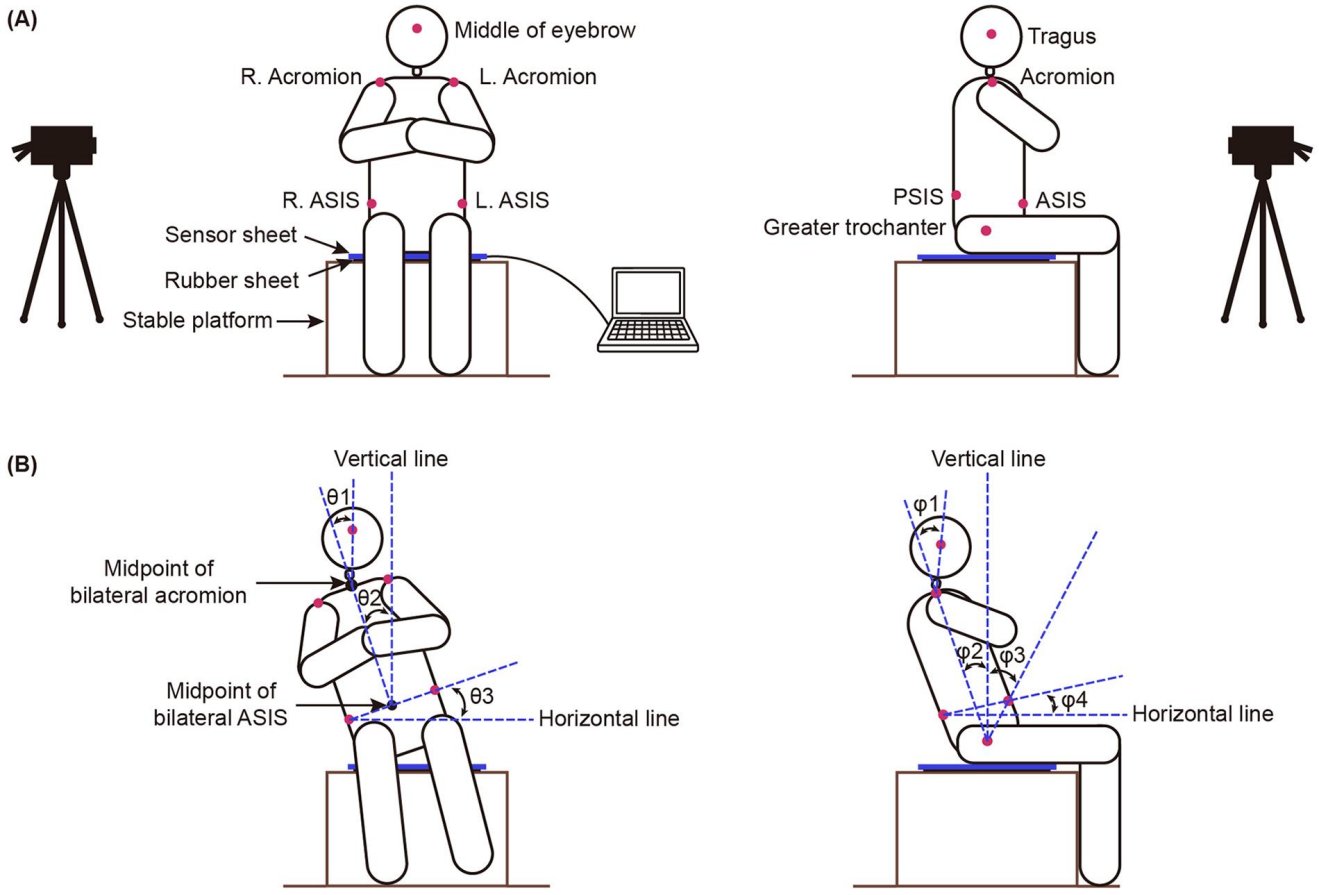
Experimental setup and body segment angles. **(A)** Experimental setup. Left and right panels show front and right-side views, respectively. **(B)** Body segment angles. Left and right panels show front and right-side views, respectively. In the front view, the head (*θ*1) and trunk (*θ*2) angles represent lateral tilt (left–right inclination), and the pelvic tilt angle (*θ*3) represents pelvic obliquity. In the right-side view, the head (*φ*1), trunk (*φ*2), and hip (*φ*3) angles represent flexion/extension (anterior–posterior inclination), and the pelvic tilt angle (*φ*4) represents anterior/posterior pelvic tilt.

Figure 1A details the experimental setup. The participants sat on a stable platform with their feet naturally positioned (approximately hip-width apart) and their arms crossed over their chests. The height of the platform was adjusted so that the knees and hips were flexed to approximately 90°.

In the experimental session, the participants were asked to voluntarily lean their trunks in four directions at a comfortable speed: left, right, forward, and backward. The participants were instructed to move their left or right shoulder, forehead, or occiput as far as possible. Movement quality was monitored in real time, and trials were repeated if obvious axial rotation of the trunk was observed. After reaching maximum leaning, they paused for approximately two seconds and returned to the starting upright sitting position. While leaning, participants were asked to keep at least one foot on the floor. Prior to the measurement session, the participants practiced the task no more than twice in each direction to minimize potential learning effects while watching the demonstration videos. In the measurement session, to reduce the potential order effect on subsequent movement, each direction of the trunk-leaning task was performed twice sequentially in the following fixed order: backward, left, right, and forward in the first trial (Trial 1) and right, backward, forward, and left in the second trial (Trial 2).

Leaning movements were captured using two video cameras positioned approximately 1.5 m from the front and right sides of the participant (sampling frequency: 30 Hz). To precisely label the position of the anatomical landmarks, eight color markers with approximately 2.0 cm in diameter were attached to the middle of the eyebrow, left and right acromion, left and right anterior superior iliac spine (ASIS), right tragus, right posterior superior iliac spine (PSIS), and right greater trochanter (Figure 1A).

To measure COP displacement, the pressure distribution on the buttocks was measured using a sensor sheet (Smart Rubber Soft Vision, Numerical Version, Sumitomo Riko, Aichi, Japan) placed under the buttocks on the platform. The sheet consisted of 256 pressure-detection sensors (16 × 16 sensors) distributed over an area of 350 mm × 350 mm, with each sensor spaced 2 mm apart (Supplementary Material 1; Supplementary Figure S1). This sensor sheet has been used in previous studies examining pressure distribution and COP-related metrics while sitting (21, 22), and its performance characteristics have been assessed under standardized bench-testing conditions (23). Data from the sensor sheet were sampled at a frequency of 5 Hz and stored on a laptop, consistent with a previous study (21). A 5-mm-thick rubber sheet was placed between the sensor sheet and the plywood surface of the platform to prevent the sensor sheet from sliding.

### 2.3 Data analysis

#### 2.3.1 COP displacement

The COP coordinates (X_center_, Y_center_), representing the spatial coordinates of the sensor sheet, were calculated based on the values measured by the 256 sensors using the following formulae (24):

$$X_{center}=\frac{\sum_{x=0}^{15}\sum_{y=0}^{15}xP_{x,y}}{\sum_{x=0}^{15}\sum_{y=0}^{15}P_{x,y}}\;\;\;\;\;$$ (1)

$$\;Y_{center}=\frac{\sum_{x=0}^{15}\sum_{y=0}^{15}yP_{x,y}}{\sum_{x=0}^{15}\sum_{y=0}^{15}P_{x,y}}$$ (2)

X_center_ and Y_center_ represent the x- and y-coordinates of the COP, respectively, and were calculated by dividing the sum of the products of each sensor's x-coordinate or y-coordinate and its corresponding pressure value by the sum of all pressure values from the sensors (Supplementary Material 1; Supplementary Figure S2). Based on the time-course changes in the COP coordinates during the leaning task, we measured the maximum distance of COP displacement from the location where the COP started to move in each direction using a custom-made MATLAB program (MathWorks, Inc., Natick, MA, USA). The displacement was calculated as the movement distance in the x-coordinate for left and right leanings and in the y-coordinate for forward and backward leanings. We then computed the unsigned sum of the displacements in the left and right directions, as well as those in the forward and backward directions, as a proxy for the COP displacements in the left-right and forward-backward directions, respectively. COP displacement was calculated for Trials 1 and 2.

#### 2.3.2 Changes in body segment angles

The spatial positions of the body segments were analyzed using the open-source software DeepLabCut (DLC; Version 2.3.5, Mathis Group & Mathis Lab, Swiss Federal Institute of Technology, Lausanne, Switzerland) (25). DLC-based 2D kinematic analyses have been applied to large-amplitude human movements with acceptable pixel-level errors (26), and have also been validated against gold-standard marker-based 3D motion capture systems, demonstrating good-to-excellent agreement for joint angle measurements (27, 28). This analysis was based on the methods described in earlier studies (29, 30) and utilized videos captured during the leaning task. For the DLC measurement, 40 frames were automatically extracted from each video using OpenCV's K-means algorithm. Specific body segments were then labeled with circular dots of different colors. Five anatomical landmarks were labeled from the frontal-view video: the middle of the eyebrow, left acromion, right acromion, left ASIS, and right ASIS. Five additional landmarks were labeled from the sagittal view (from the right side) video: the tragus, acromion, ASIS, PSIS, and greater trochanter. After labeling the extracted frames, the training process saved the results every 5,000 iterations and continued until a total of 500,000 iterations were completed. These iterations contributed to refining the model's accuracy for detecting and tracking labeled landmarks, ensuring a reliable spatial analysis of body segment movements.

Based on the spatial coordinates of each landmark estimated by DLC, changes in the body angles during the leaning task were calculated using a custom-made MATLAB program. The relative segment angles (i.e., not anatomical angles) of the head, trunk, and pelvic tilt in the front view and those of the head, trunk, hip, and pelvic tilt in the right-side view were defined based on previous studies (Figure 1B) (31–34). In the front view, the head (*θ*1) and trunk (*θ*2) angles represent lateral tilt (left–right inclination), and the pelvic tilt angle (*θ*3) represents pelvic obliquity. In the right-side view, the head (*φ*1), trunk (*φ*2), and hip (*φ*3) angles represent flexion/extension (anterior–posterior inclination), and the pelvic tilt angle (*φ*4) represents anterior/posterior pelvic tilt. The time-course data of body angles were first smoothed by applying a second-order Butterworth low-pass filter with a cutoff frequency of 6 Hz (35). Body segment angle changes were treated as signed values: movements in the same direction as the instructed leaning direction were defined as positive, whereas movements in the opposite direction were defined as negative. Subsequently, for the left and right leanings, the maximal angle changes of each body segment were measured by calculating the sum of angles during the left and right leanings at the endpoint when the left or right shoulder moved the farthest. Similarly, for the forward and backward leanings, the maximal angle changes of each body segment were calculated as the sum of angles during the forward and backward leanings at the endpoint when the right shoulder moved the farthest in the forward or backward directions. Body angle changes were calculated for Trials 1 and 2.

#### 2.3.3 Reliability of the measured indices

First, we examined the within-session reliability of the measured indices. The differences between the two trials of the measured indices were tested for normal distribution using the Shapiro–Wilk test. After confirming a normal distribution, the intraclass correlation coefficients (ICCs) (3,1) and 95% confidence intervals (CI) were calculated between the two trials to estimate the relative reliability. The strength of agreement (i.e., correlation) was interpreted as follows: <0.00, slight; 0–0.19, low; 0.20–0.39, moderate; 0.40–0.69, high; 0.70–0.89, substantial; and 0.90–1.00, very high (36).

To examine the absolute reliability and estimate the limits of agreement (LOA) between the two trials, Bland–Altman analyses were performed on the measured indices. The presence of fixed bias was confirmed when the 95% CI of the mean differences between the two trials ($\bar{d}$) did not include zero, and the proportional bias was tested using linear regression analysis to confirm if the difference between the two trials (d or |d|) was significantly correlated with the mean of the two trials (37). The LOA was calculated as the mean ± 1.96 standard deviations (SDs) of the differences. The 95% CIs of the upper and lower LOAs were also calculated (38). In addition, the minimal detectable change (MDC) with a 95% CI (MDC_95_) was calculated using the standard error of measurement (SEM) to quantify the measurement errors.

$$\mathrm{MD}C_{95}\;=\;1.96\;\mathrm{SEM}\;\;\times \;\sqrt{2}$$ (3)

$$\mathrm{MD}C_{95}\text{\%}\;=\;(\mathrm{MD}C_{95}\;/\;\mathrm{mean}\;\mathrm{of}\;\mathrm{two}\;\mathrm{trials})\;\times \;100$$ (4)

In this study, we adopted an SEM calculation method based on the SD of the differences between two trials (SDd), as previously described (39, 40):

$$\mathrm{SEM}\;=\;\mathrm{SDd}\;/\;\sqrt{2}\;$$ (5)

#### 2.3.4 Association between each body segment movement and COP displacement

To evaluate the associations between each body segment movement (i.e., angle changes) and COP displacements, we performed multivariable mixed-effects models separately for left–right leaning and forward–backward leaning. For left–right leaning, mediolateral COP displacement was included as the dependent variable, while changes in each of head, trunk, and pelvic tilt angles were included individually as explanatory variables in separate models. For forward–backward leaning, anteroposterior COP displacement was included as the dependent variable, while changes in each of head, trunk, hip, and pelvic tilt angles were included individually as explanatory variables in separate models. In these multivariable models, each segment angle change was included as fixed effect, and the number of trials and height were included as additional fixed effects to adjust for the potential confounding factor of COP displacement (20). Participant was included as a random effect.

The statistical significance of each body segment variable was assessed using the *p*-values of its corresponding fixed-effect coefficients (slope parameters) in each mixed-effects model.

#### 2.3.5 Statistical analyses

All statistical analyses were conducted using SPSS version 26 (IBM Corp., Armonk, NY, USA). The effects were considered significant if *p* was <0.05.

### 2.4 Reporting guideline

We used the Strengthening the Reporting of Observational Studies in Epidemiology (STROBE) reporting guideline (41) to draft this manuscript, and the STROBE reporting checklist (42) when editing, included in Supplementary Material 2.

## 3 Results

### 3.1 Reliability of changes in body segment angles and COP displacements

**Figure 2:**
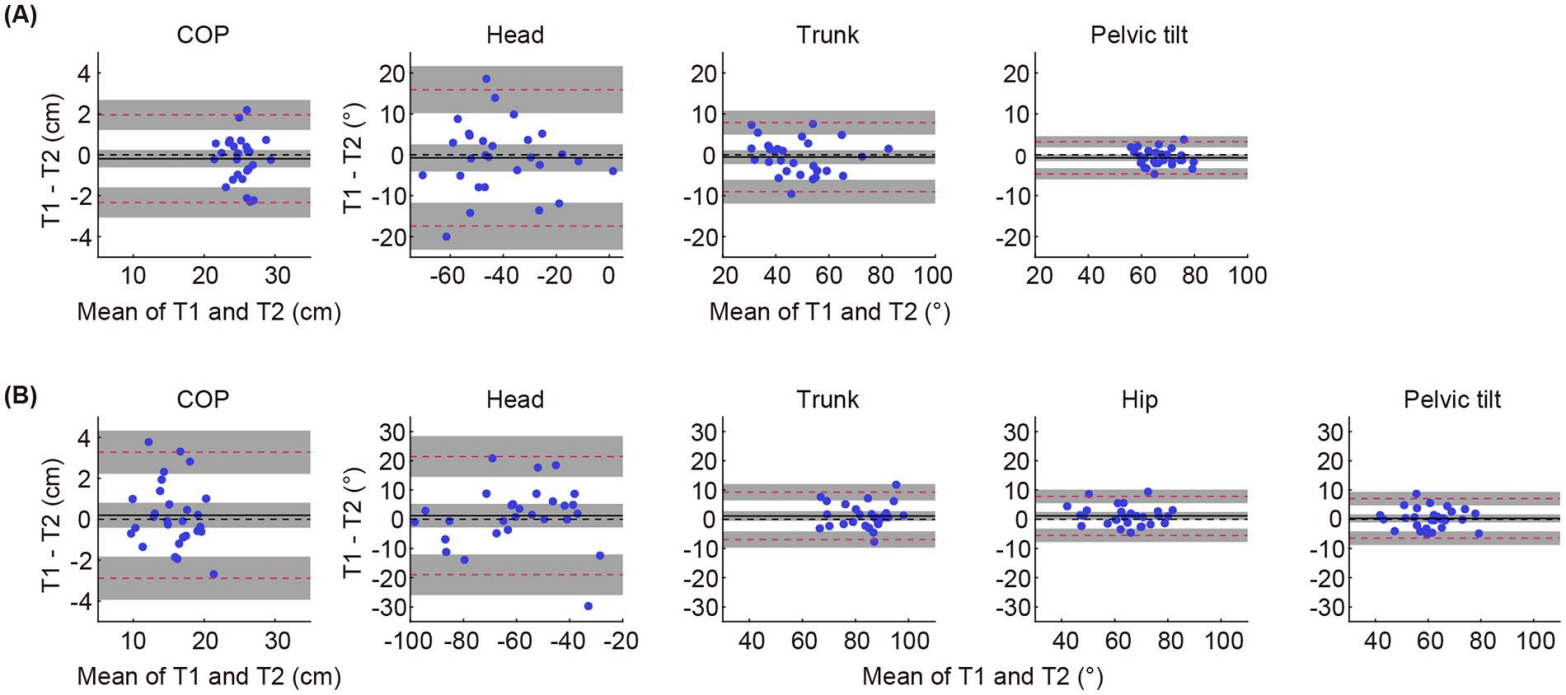
Bland–Altman plots for COP displacements and changes in body segment angles. **(A)** Left-right lean. **(B)** Forward-backward lean. *X*-axis values represent the mean COP displacements/body segment angle changes of T1 and T2. *Y*-axis values represent the difference between the trials. The black solid horizontal lines represent the mean of the difference between the trials. The black dashed horizontal lines represent zero. The red dashed lines represent the upper and lower 95% LOAs. The shaded areas represent the 95% confidence intervals for the mean and LOAs. Each dot represents individual data. COP, center of pressure; T1, Trial 1; T2, Trial 2; LOA, limit of agreement.

Normal distribution was confirmed for the data of the measured indices. The relative reliability [ICC (3,1)] between the two trials confirmed a substantial to very high correlation among all indexes (ICC = 0.84–0.95) (Table 2). Furthermore, the Bland–Altman plots showed neither significant fixed nor proportional biases in the COP displacements or angle changes of each body segment over the two trials (Figure 2; Table 2), suggesting sufficient absolute reliability of the measured indexes. During left-right leaning, the MDC_95_ values for COP displacement and head, trunk, and pelvic tilt angle changes between trials were 2.1 cm, 16.7°, 8.5°, and 3.9°, respectively. During forward-backward leaning, the MDC_95_ values for the COP displacement and head, trunk, hip, and pelvic tilt angle changes between trials were 3.1 cm, 20.2°, 8.1°, 6.7°, and 6.7°, respectively (Table 2). When qualitatively comparing the characteristics of the body segment angle changes, we found that the MDC_95_% in the head angle changes between Trials 1 and 2 tended to be larger than those of the other body segments (Table 2).

**Table 2:** Reliability of changes in body segment angles and center of pressure (COP) displacements.

Analysis	Parameter		COP	Head	Trunk	Hip	Pelvic tilt
Relative reliability							
Left-right lean							
ICC			0.84 (0.69, 0.92)	0.88 (0.76, 0.94)	0.95 (0.89, 0.97)	–	0.95 (0.90, 0.98)
Forward-backward lean							
ICC			0.89 (0.78, 0.95)	0.87 (0.74, 0.94)	0.90 (0.79, 0.95)	0.95 (0.89, 0.98)	0.94 (0.87, 0.97)
Absolute reliability							
Left-right lean							
Fixed bias	$\bar{d}$		−0.2 (−0.61, 0.23)	−0.8 (−4.05, 2.55)	−0.6 (−2.23, 1.11)	–	−0.7 (−1.50, 0.04)
	Lower LOA		−2.3 (−3.07, −1.60)	−17.4 (−23.16, −11.72)	−9.0 (−11.91, −6.12)	–	−4.6 (−5.97, −3.30)
	Upper LOA		2.0 (1.22, 2.69)	16.0 (10.22, 21.65)	7.9 (5.00, 10.79)	–	3.2 (1.84, 4.52)
Proportional bias	d vs. mean	r	0.11	0.03	0.16	–	0.09
		p	0.580	0.881	0.415	–	0.654
	|d| vs. mean	r	0.16	0.21	0.04	–	0.08
		p	0.409	0.283	0.854	–	0.679
Forward-backward lean							
Fixed bias	$\bar{d}$		0.2 (−0.41, 0.81)	1.3 (−2.75, 5.25)	1.1 (−0.46, 2.75)	1.2 (−0.15, 2.48)	0.3 (−1.03, 1.64)
	Lower LOA		−2.9 (−3.94, −1.82)	−19.0 (−25.92, −12.05)	−7.0 (−9.73, −4.18)	−5.5 (−7.76, −3.20)	−6.4 (−8.75, −4.13)
	Upper LOA		3.3 (2.22, 4.33)	21.5 (14.55, 28.42)	9.3 (6.47, 12.03)	7.8 (5.54, 10.10)	7.1 (4.74, 9.36)
Proportional bias	d vs. mean	r	0.20	0.05	0.03	0.14	0.04
		*p*	0.319	0.820	0.900	0.489	0.858
	|d| vs. mean	r	0.02	0.22	0.10	0.13	0.01
		*p*	0.908	0.251	0.621	0.500	0.966
Minimal detectable changes							
Left-right lean							
SEM			0.77	6.02	3.05	–	1.41
MDC_95_			2.1	16.7	8.5	–	3.9
MDC_95_%			8.5	41.0	17.5	–	5.8
Forward-backward lean							
SEM			1.11	7.30	2.92	2.40	2.43
MDC_95_			3.1	20.2	8.1	6.7	6.7
MDC_95_%			19.7	34.0	9.7	10.4	11.1

Values in parentheses indicate 95% confidence intervals. ICC, intraclass correlation coefficient; LOA, limit of agreement; SEM, standard error of measurement; MDC_95_, minimal detectable change with a 95% confidence interval.

### 3.2 Association between angle changes of each body segment and COP displacements

**Figure 3:**
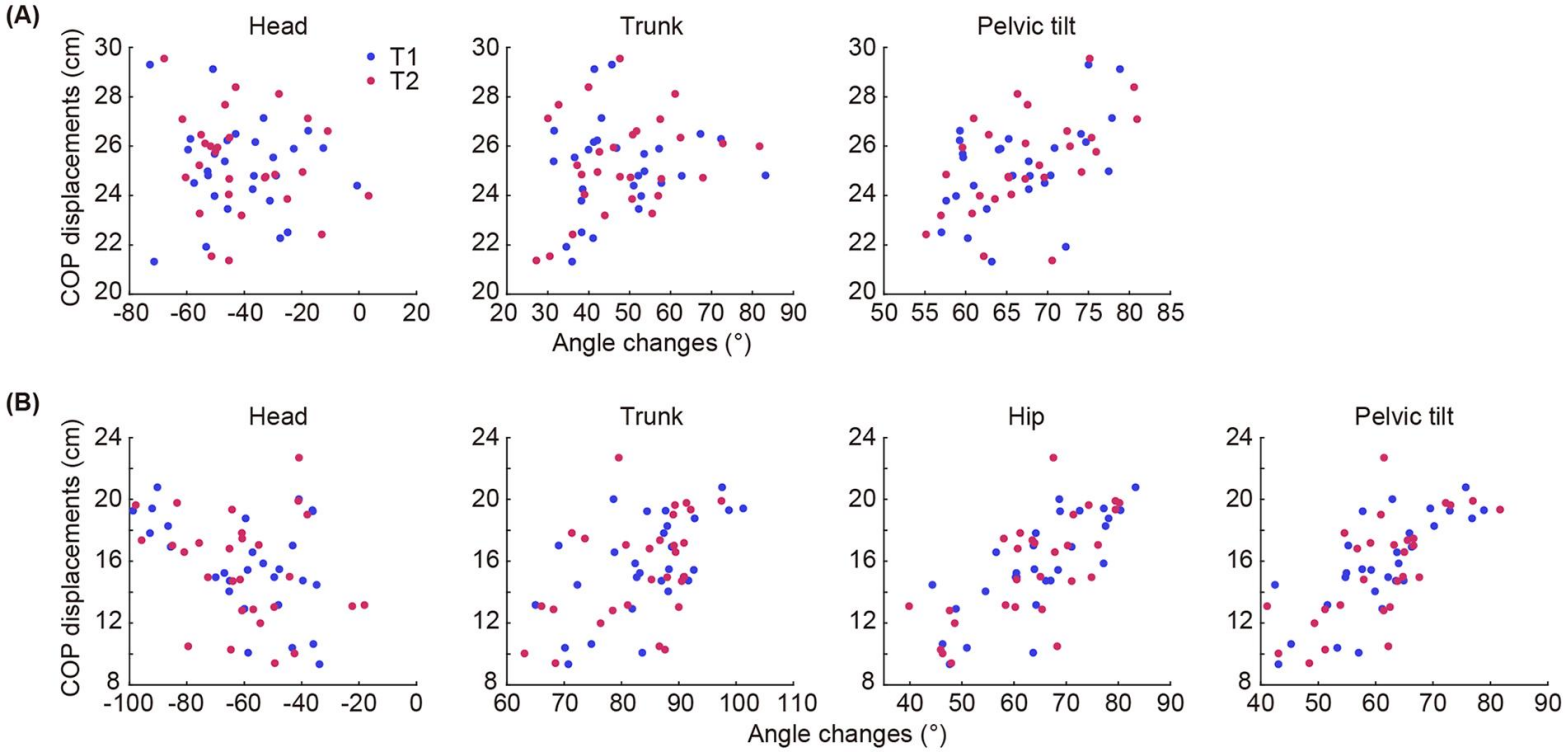
Scatter plots depicting changes in body segment angles and COP displacements. **(A)** Left-right lean. **(B)** Forward-backward lean. *X*-axis values represent angle changes for each body segment. *Y*-axis values represent COP displacements. The red and blue dots represent individual data from T1 and T2, respectively. COP, center of pressure; T1, Trial 1; T2, Trial 2.

 Figure 3 illustrates the relationship between the angle changes in each body segment and the COP displacements. Table 3 provides the results of the mixed-effects modeling of the COP displacements. Changes in the pelvic tilt angle were significantly associated with the COP displacements during the left-right and forward-backward leans (*p* < 0.001). In addition, changes in head, trunk, and hip angles were significantly associated with COP displacement during forward-backward leaning (all *p* < 0.01). Note that the head moved in the opposite direction of the trunk lean (minus values of angle changes) in almost all participants, regardless of the leaning direction (Figure 3).

**Table 3:** Mixed-effects model analyses for the association between body segment angle changes and center of pressure displacements.

Directions	Parameter	Head	Trunk	Hip	Pelvic tilt
Left-right lean	*β*	−0.03 (−0.06, 0.00)	0.04 (−0.01, 0.09)	–	0.18 (0.09, 0.26)
	*p*-value	0.064	0.121		<0.001
Forward-backward lean	β	−0.07 (−0.11, −0.03)	0.19 (0.10, 0.28)	0.25 (0.18, 0.31)	0.25 (0.17, 0.33)
	*p*-value	0.001	<0.001	<0.001	<0.001

Values in parentheses indicate 95% confidence intervals.

Specifically, in left-right leaning, a 1° increase in pelvic obliquity change was associated with approximately 0.18 cm larger COP displacement (*p* < 0.001). In forward-backward leaning, a 1° increase in pelvic tilt was associated with approximately 0.25 cm larger COP displacement, and comparable associations were also observed for trunk (0.19 cm/°) and hip (0.25 cm/°) angle changes (all *p* < 0.001). Head angle change showed a significant negative association with COP displacement (−0.07 cm/°) (*p* < 0.01) (Table 3).

## 4 Discussion

There has been limited systematic quantification of COP displacements and body segmental strategies during seated trunk leaning. Understanding of a quantitative baseline of these metrics during a standardized trunk leaning task in healthy adults provides a reference for interpreting abnormal strategies and for evaluating interventions in clinical and aging populations. This study investigated the relationship between changes in body segment angles and COP displacements during trunk leaning movements while sitting. The results revealed that the angle change of the pelvic tilt was significantly associated with COP displacement during trunk leaning, regardless of the direction. This study's results offer valuable insights into postural control strategies for maintaining sitting balance in healthy adults, indicating the importance of pelvic control in COP shifts during sitting.

To confirm the reliability of the measured indices, we first investigated the within-session reliability of the COP displacements and angular changes in the body segments. The ICC values ranged from 0.84 to 0.95, indicating substantial to very high correlations between the two trials. Furthermore, the Bland–Altman analyses showed neither fixed nor proportional biases. Taken together, these results indicated that our measurements had sufficient relative and absolute reliability.

Our primary results revealed that changes in the head, trunk, hip, and pelvic tilt angles were significantly associated with COP displacement in forward-backward leaning, but only the pelvic tilt angle had a significant relationship with COP displacement in left-right leaning. This result highlights the notable association between pelvic tilt and COP displacement, regardless of the leaning direction. The pelvis is an important element for maintaining standing and sitting postures and balance (43). It connects the spine and lower limbs and enhances body stability by providing support to the upper body (44). A previous study demonstrated that during maximal lateral reaching, pelvic tilt in the frontal plane is required in healthy participants, indicating the dynamic role of the pelvis in extending COP displacement (20). Other studies on individuals with pathologies have suggested that the pelvis plays an essential role in effective postural control. Kinematic measurements during seated leaning have identified lateral pelvic movement as the best predictor of improvement in trunk motor function and a major contributor to sitting balance after stroke (45). In addition, post-stroke individuals demonstrated trunk flexion with an amplitude comparable to that of healthy controls, but they exhibited a limited amount of COP displacement, likely due to slight movement of the anterior pelvic tilt (46). These studies suggest that impaired pelvic movement may be associated with reduced COP control. To our knowledge, ours is the first study to systematically investigate the segmental associations between each body part and COP displacement during leaning in multiple directions in healthy adults and to statistically quantify the association between pelvic movement and COP control. Although caution should be exercised when interpreting the results, because COP displacement reflects whole-body dynamics [i.e., changes in the center of body mass [COM], a weighted average of the COMs of each body segment (47)] rather than isolated segmental effects, the findings in this study may provide a reference for understanding the underlying mechanisms of postural control under pathological conditions.

One potential explanation why all body segments were associated with the COP displacements in forward-backward leaning—differing from left-right leaning, where only pelvic tilt showed a significant relationship—may be related to differences in the available support between directions. In our task, participants sat on the sensor sheet and were instructed to keep at least one foot on the floor during leaning. The thighs, feet, and buttocks may provide greater support for the forward-backward leaning. Studies have found that providing foot support increases the maximum forward COP displacement compared to conditions without foot support (48, 49). This larger base likely offers greater stability and may allow coordinated engagement of all body segments to lean forward without losing balance. In contrast, left-right leaning involves lateral weight shifting within a limited base of support. This can explain our findings that the COP may be associated with a significant pelvic tilt to maximize its displacement, with less association with supportive trunk movement. Future work integrating surface electromyography (e.g., paraspinal and abdominal muscles) would strengthen the mechanistic interpretation by linking the kinematic strategies to underlying neuromuscular activation patterns.

Another important finding regarding body segment control was the specific movement of the head. In this study, the head moved in the opposite direction of the trunk lean in almost all participants, regardless of the leaning direction. This countermovement may be a balance-maintaining mechanism. Previous research has highlighted the head's significant role in eliciting and modifying postural responses regulated by the vestibulocollic, cervicocollic, and visual reflexes (50–52). Similarly, head movements in the contralateral direction were observed in healthy participants during seated arm-reaching tasks (53). Such a counter-response appears to be an important strategy for maintaining balance by keeping the COM close to, yet within, the border of the base of support. To confirm the contribution of the COM to the association between the COP and the body segment movements, future studies will be needed. Moreover, there was a significant association between head angle changes and COP displacements in forward-backward leaning. It means that greater head countermovement was associated with greater COP displacement. This also suggests that the head-neck complex may act as a stabilization strategy, which allows us to increase COP displacements during seated leaning movements. This behavior may involve anticipatory postural adjustments and/or compensatory mechanisms. However, because the present study did not examine the temporal onset of segmental movements or muscle activity, we cannot distinguish between these possibilities. Besides, in the present study, the magnitude of head countermovement exhibited greater trial-to-trial variability compared to the other body segments, as reflected by the larger MDC_95_% of the head angle changes between Trials 1 and 2 compared to those of other body segments. This finding indicated that, while the direction of head countermovement represents a consistent balance-maintaining strategy, the magnitude of this motion may involve adaptive or exploratory adjustments in association with sitting balance control and thus may not always be fixed across trials. The relatively large trial-to-trial variability in head movements may reflect the head's role as a flexible postural adjuster—positioned distally relative to the base of support—in maintaining dynamic sitting balance.

This study had several limitations. First, it's worth noting that the separate modeling strategy we performed may not fully account for interaction effects of segments and may leave potential residual confounding or omitted-variable bias. We did not include all body segments simultaneously in the mixed-effects model to avoid potential risk of overfitting, due to the small sample size (54). In addition, there was a risk of multicollinearity because segmental movements during trunk leaning are mechanically linked and may not represent fully independent variables. Second, our data set did not include body weight and trunk height in the analysis, which may reduce the biomechanical precision of the interpretation. Third, this study only included healthy young adults. Previous studies have shown that older adults and post-stroke individuals have smaller reaching distances and lower COP displacements compared to younger adults (5, 53, 55). Changes in sensory, musculoskeletal, and cognitive functions in older adults and individuals with stroke may affect movement strategies during seated leaning. Applying the present standardized, multidirectional seated trunk leaning task to a wider age range and to neurological disorders could provide further insights into the different strategies of each body segment for COP control across populations and may help translate these mechanistic insights into clinically meaningful benchmarks. Fourth, although the present analysis focused on maximum COP displacement during a voluntary, relatively slow trunk-leaning task, with an approximately two-second maximum leaning hold, the low sampling frequency of 5 Hz for COP measures may ignore small positional changes derived from rapid transient COP dynamics such as oscillations while holding still. Finally, our pose estimation analysis was two-dimensional; any out-of-plane movement or axial rotation during trunk leaning may introduce projection error, leading to inaccurate estimation of segmental angles. Such measurement error may affect the strength of associations with COP. Although we attempted to minimize this issue by using standardized demonstration videos and monitoring participants’ performance in real time, residual out-of-plane movements may not have been fully eliminated. Future studies using three-dimensional motion analysis are needed to quantify rotational components more accurately.

## 5 Conclusions

This study's results suggest that changes in the pelvic tilt angle were particularly associated with COP displacements during trunk leaning movements, regardless of direction, in healthy adults. Although these findings are correlational and exploratory, this normative dataset may provide a useful reference for understanding impaired sitting balance in older adults and neurological populations.

## Data Availability

The raw data supporting the conclusions of this article will be made available upon reasonable request.
