# Supplementary material for: Relationship between body segment movements and center of pressure shifts during trunk lean movements while sitting in healthy adults
Source: Front Rehabil Sci. 2026 Jun 10;7:1837819. doi: 10.3389/fresc.2026.1837819 (PMC13290909; doi:10.3389/fresc.2026.1837819)
Supplement: Supplementary file 1 [file Datasheet1.pdf]

Supplementary Material 1

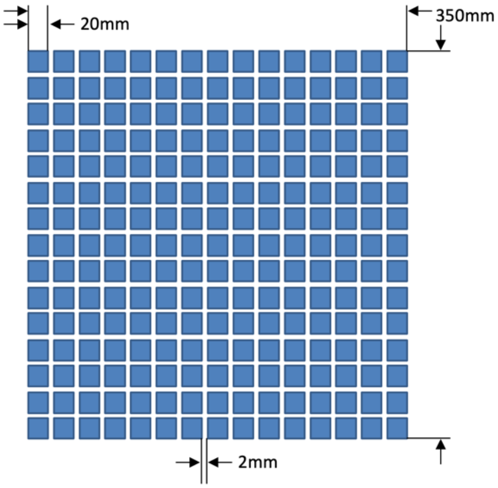

Figure S1. Pressure sensor distribution. The sheet consists of 256 sensors (16 × 16 sensors) distributed over a 350 mm × 350 mm area with 2 mm between each sensor.

Y \ X	0	1	2	3	4	5	6	7	8	9	10	11	12	13	14	15	
0	0	0	0	0	0	50	60	70	60	50	0	0	0	0	0	0	2030
1	0	0	0	0	0	60	70	80	70	60	0	0	0	0	0	0	2380
2	0	0	0	0	0	70	80	90	80	70	0	0	0	0	0	0	2730
3	0	0	0	0	0	60	70	80	70	60	0	0	0	0	0	0	2380
4	0	0	0	0	0	50	60	70	60	50	0	0	0	0	0	0	2030
5	0	0	0	0	0	0	0	0	0	0	0	0	0	0	0	0	0
6	0	0	0	0	0	0	0	0	0	0	0	0	0	0	0	0	0
7	0	0	0	0	0	0	0	0	0	0	0	0	0	0	0	0	0
8	0	0	0	0	0	0	0	0	0	0	0	0	0	0	0	0	0
9	0	0	0	0	0	0	0	0	0	0	0	0	0	0	0	0	0
10	0	0	0	0	0	0	0	0	0	0	0	0	0	0	0	0	0
11	0	0	0	0	0	0	0	0	0	0	0	0	0	0	0	0	0
12	0	0	0	0	0	0	0	0	0	0	0	0	0	0	0	0	0
13	0	0	0	0	0	0	0	0	0	0	0	0	0	0	0	0	0
14	0	0	0	0	0	0	0	0	0	0	0	0	0	0	0	0	0
15	0	0	0	0	0	0	0	0	0	0	0	0	0	0	0	0	0
	0	0	0	0	0	580	680	780	680	580	0	0	0	0	0	0	1650

Sum	1650
XSum	11550
YSum	3300

X_{center}	7
Y_{center}	2

Example

50	60	70	60	50
60	70	80	70	60
70	80	90	80	70
60	70	80	70	60
50	60	70	60	50

$$Sum = \sum_{x=0}^{15} \sum_{y=0}^{15} P_{x,y}$$

$$X\,Sum = \sum_{x=0}^{15} \sum_{y=0}^{15} xP_{x,y}$$

$$Y\,Sum = \sum_{x=0}^{15} \sum_{y=0}^{15} yP_{x,y}$$

$$X_{center} = \frac{X\ Sum}{Sum}$$

$$Y_{center} = \frac{Y\ Sum}{Sum}$$

Figure S2. Example computations for the X_{center} and Y_{center} coordinates. X_{center} and Y_{center} represent the x-coordinate and y-coordinate of the center of pressure, respectively. These coordinates are computed by taking the sum of the products of each sensor's x or y coordinates and the corresponding pressure value, divided by the total sum of all pressure values from the sensors.
