# Supplementary material for: Relationship between body segment movements and center of pressure shifts during trunk lean movements while sitting in healthy adults
Source: Front Rehabil Sci. 2026 Jun 10;7:1837819. doi: 10.3389/fresc.2026.1837819 (PMC13290909; doi:10.3389/fresc.2026.1837819)
Supplement: Supplementary file 2 [file Datasheet2.pdf]

Supplementary Material 2

The STROBE reporting checklist

	Item Description	Location (or reason for not reporting)
**Title and abstract**		
1a. Indicate the study's design	Indicate the study's design with a commonly used term in the title or the abstract.	N/A; we didn't state the specific study design since it's not a clinical study.
1b. Abstract	Provide in the abstract an informative and balanced summary of what was done and what was found.	Abstract.
**Introduction**		
2. Background / rationale	Explain the scientific background and rationale for the investigation being reported.	Introduction, paragraphs 1 & 2.
3. Objectives	State specific objectives, including any prespecified hypotheses.	Introduction, paragraph 3.
**Methods**		
4. Study design	Present key elements of study design early in the paper.	Methods, Participants characteristics; Methods, Experimental procedure.<br>We didn't state the specific study design since it's not a clinical study.
5. Setting	Describe the setting, locations, and relevant dates, including periods of recruitment, exposure, follow-up, and data collection.	Methods, Participants characteristics; Methods, Experimental procedure.<br>No exposure or follow-up period was applicable.
6a. Eligibility criteria	**Cohort study:** Give the eligibility criteria, and the sources and methods of selection of participants. Describe methods of follow-up. **Case-control study:** Give the eligibility criteria, and the sources and methods of case ascertainment and control selection. Give the rationale for the choice of cases and controls. **Cross-sectional study:** Give the eligibility criteria, and the sources and methods of selection of participants.	Methods, Participants characteristics.

6b. Matching criteria	**Cohort study:** For matched studies, give matching criteria and number of exposed and unexposed. **Case-control study:** For matched studies, give matching criteria and the number of controls per case.	N/A; this was not a cohort or case-control study.
7. Variables	Clearly define all outcomes, exposures, predictors, potential confounders, and effect modifiers. Give diagnostic criteria, if applicable.	Methods, Data analysis, sections 1-4.<br>No diagnostic criteria were applicable because this study included healthy adults.
8. Data sources / measurement	For each variable of interest give sources of data and details of methods of assessment (measurement). Describe comparability of assessment methods if there is more than one group.	Methods, Experimental procedure; Methods, Data analysis, sections 1-2. Figure 1, S1, S2.<br>Comparability of assessment methods was not applicable.
9. Bias	Describe any efforts to address potential sources of bias.	Methods, Experimental procedure; Discussion, paragraph 6.
10. Study size	Explain how the study size was arrived at.	Methods, Participants characteristics.
11. Quantitative variables	Explain how quantitative variables were handled in the analyses. If applicable, describe which groupings were chosen, and why.	Methods, Data analysis, sections 1-4.
12a. Statistical methods	Describe all statistical methods, including those used to control for confounding.	Methods, Data analysis, sections 3-5.
12b. Statistical methods – subgroups and interactions	Describe any methods used to examine subgroups and interactions.	N/A; subgroup and interaction analyses were not performed in this study.
12c. Statistical methods – missing data	Explain how missing data were addressed.	N/A; no missing data were present for the variables included in the final analysis.
12di. Statistical methods – loss to follow-up	**Cohort study:** If applicable, describe how loss to follow-up was addressed.	N/A; this was not a cohort study and did not involve follow-up.
12dii. Statistical methods – matching cases and controls	**Case-control study:** If applicable, explain how matching of cases and controls was addressed.	N/A; this was not a case-control study.

12diii. Statistical methods – sampling strategy	**Cross-sectional study:** If applicable, describe analytical methods taking account of sampling strategy.	N/A; no sampling strategy was used in this study.
12e. Statistical methods – sensitivity analyses	Describe any sensitivity analyses.	N/A; sensitivity analyses were not performed in this study.
**Results**		
13a. Participant numbers	Report the numbers of individuals at each stage of the study—e.g., numbers potentially eligible, examined for eligibility, confirmed eligible, included in the study, completing follow-up, and analysed; Consider use of a flow diagram.	Methods, Participants characteristics; Table 1.
13b. Participants – non-participation	Give reasons for non-participation at each stage.	Methods, Participants characteristics.
13c. Participants – flow diagram	Consider use of a flow diagram.	N/A; a flow diagram was not used because the participant flow in this study was simple and was described in the Participants characteristics section.
14a. Descriptive data – participant characteristics	Give characteristics of study participants (e.g., demographic, clinical, social) and information on exposures and potential confounders. Present the information in a table.	Table 1.
14b. Descriptive data – missing data	Indicate the number of participants with missing data for each variable of interest.	N/A; no missing data were present for the variables included in the final analysis.
14c. Descriptive data – follow-up time	**Cohort study:** Summarise follow-up time—e.g., average and total amount.	N/A; this was not a cohort study, and no follow-up time was assessed.
15. Outcome data	**Cohort study:** Report numbers of outcome events or summary measures over time. **Case-control study:** Report numbers in each exposure category, or summary measures of exposure. **Cross-sectional study:** Report numbers of outcome events or summary measures.	Results; Tables 2 and 3; Figures 2 and 3.
16a. Main results	Give unadjusted estimates and, if applicable, confounder-adjusted estimates and their precision (e.g., 95% confidence intervals). Make clear which confounders were adjusted for and why they were included.	Results, Association between angle changes of each body segment and COP displacements; Table 3.

16b. Main results – category boundaries	Report category boundaries when continuous variables were categorised.	N/A; continuous variables were not categorized in this study.
16c. Main results – risk	If relevant, consider translating estimates of relative risk into absolute risk for a meaningful time period.	N/A; this study did not estimate relative or absolute risk.
17. Other analyses	Report other analyses done—e.g., analyses of subgroups and interactions, and sensitivity analyses.	N/A; no subgroup, interaction, or sensitivity analyses were performed in this study.
**Discussion**		
18. Key results	Summarise key results with reference to study objectives.	Discussion, paragraph 1.
19. Limitations	Discuss limitations of the study, taking into account sources of potential bias or imprecision. Discuss both direction and magnitude of any potential bias.	Discussion, paragraph 6.
20. Interpretation	Give a cautious overall interpretation considering objectives, limitations, multiplicity of analyses, results from similar studies, and other relevant evidence.	Discussion, paragraphs 2-5.
21. Generalisability	Discuss the generalisability (external validity) of the study results.	Discussion, paragraphs 1 & 6; Conclusions.
**Other information**		
22. Funding	Give the source of funding and the role of the funders for the present study and, if applicable, for the original study on which the present article is based.	Funding. The funders had no role in study design, data collection and analysis, decision to publish, or preparation of the manuscript.
